# Frontiers in Pain Research: A Scope of Its Focus and Content

**DOI:** 10.3389/fpain.2020.601528

**Published:** 2020-10-29

**Authors:** Tony L. Yaksh

**Affiliations:** Department of Anesthesiology and Pharmacology, University of California, San Diego, San Diego, CA, United States

**Keywords:** acute to chronic pain, analgesics, animal models, human trials, orphan pain states, opioids, neonatal-pediatri pain, veterinary pain therapeutics

Rare is the medical specialty in which the associated disease and pathology is not associated with pain and suffering and which leads inevitably to a diminished quality of life.

Consider the impact of every diagnosis ending in “-itis,” or neuropathic: from acute myositis, to chronic pancreatitis and arthritis, the impact of pain is beyond ready appreciation. Curing the disease, correcting the pathology, is the prime clinical imperative. However, it has been estimated that ~20% of the adults in Europe (1) and the US reported a chronic pain condition (2), with monetary costs for managing these conditions approaching €200 billion in Europe (3) and US$635 billion in the United States (4). Further, given the deleterious effects of pain upon the physiological and psychological state of the human or animal, managing the attendant pain and suffering is arguably of equal import. “*Divinum est opus sedare dolorem*” (Hippocrates).

While great strides have been made in our understanding of the biology and systems underlying the pain experience and his has yielded promising targets, we are faced with the recognition that candidate pain therapeutics face daunting challenges and a high bar for acceptance. Limited efficacy and adverse event profiles in clinical trials often account for lack of the continued development of a drug. Even those drugs which have received approval are beset by limitations. It is a common subtext that in clinical trials, significant effects may leave the patient with pain sufficient to meet the study inclusion criteria (5) and that numbers needed to treat (NTT) for a given drug/dose may range from 3 to 8 with large confidence intervals emphasizing that patients may differ substantially in their therapeutic response (6, 7). The adverse effects of drugs with identified efficacy in various acute and chronic pain states render even accepted drugs less desirable. So, arguably, the case is readily made that for all aspects of pain management, sufficiently efficacious and safer analgesic drugs/targets remain to be developed. I would like to point to several of the many overarching themes with which we are concerned. Here I would present several challenges relevant to the development of analgesic therapeutics in terms of the pain systems being targeted and the motifs to improve engagement of these target. As with a hologram, in biology, the complexity of the whole is in every part (8). Development of insights into pain mechanisms and into the behavioral, pharmacological, or physiological interventions to control pain, with minimal adverse consequences, is the guiding aim of this journal. Frontiers in Pain Research is a newly launched journal with a primary focus on pain and analgesia. As the Editor I would like to take this opportunity to provide some perspectives on important issues that the journal wishes to consider.

## Therapeutics in the Acute and Chronic Pain Transition

There is little doubt that acute inflammation and tissue injury such as a wound or incision, absent a direct nerve injury can lead to robust pain state that may reverse with resolution of the injury state. However, clinical observations and preclinical studies have revealed that these acute pain states may transition into a chronic pain condition (9), often with a neuropathic pharmacology (10, 11). The appearance of the chronic pain state represents one of the particular challenges in addressing the pain phenotype. Ongoing pain requires on going management. Clinical trial formats for addressing approaches to such hypothesized transition have been proposed (12). The challenge is the understanding of the pathological changes leading to the chronic pain condition. The now accepted phenomena raises many theoretical questions, the answers to which have practical implications. Is the transition reflective of the engagement of specific signaling systems that are addressed by specific pharmacological targets? Are these signaling systems relevant to the maintenance of the persistent pain condition or their absence lead to a chronic pain state (13, 14). To what degree does the pharmacology of the acute phase diverge from the chronic phase? What are the initiating mechanisms of a chronic state, high frequency afferent traffic, persistence of the afferent traffic? At what level(s) of the neuraxis is this chronification mediated (primary afferent, dorsal root ganglion, dorsal horn and/or brain)? What is the role of innate and adaptive immune signaling in the chronification? (15) What role does the sex of the subject play (16). Based on the above answers, one might speculate that therapeutics regulating early and late pain may not be the same as the pharmacology of the therapeutic which targets the transition (17, 18).

## The Opioid Delimma

There is little doubt that the opiate receptor and its signaling cascades are so functionally positioned as to produce a most profound regulation of nociceptive processing, whether though its ability to alter the ascending transmission of nociceptive content and/or diminish the aversive nature of the nociceptive content. The real challenge with analgesics in general and opiates in particular are 3-fold: (i) separating the role in pain modulation of this receptor from its role in reward and its endowment with properties that induce motivation to continue its administration independent of the pain state for which it might have been originally administered, (ii) the presence of tolerance (tachyphylaxis) and dependence, (iii) the phenomena of agonist (opioid) induced hyperalgesia. Whether the salutary effect and the deleterious components of opiate receptor activation can be separated represents one of the major conundrums in pain biology. Potential advances in receptor biology including biased signaling, splice variants, the role of heteromers may arguably yield covariates that serve to distinguish analgesia from the adverse aspects of the opioid receptor profile (19, 20). Development of ligands with altered receptor engagement, identification of anti-analgesic components of drug effects as for example concurrent activation of Gs coupled targets (such as the MRGs) (21) some of which are on primary afferents (22) represent important lines of research.

## Pain Therapeutics and Orphan Pathologies

It is clear that major areas of focus are those pathologies that engage large populations: post-surgical pain mononeuropathies, polyneuropathies such as present in diabetes and chemotherapy. Yet there are many examples where relatively rare pathological states are associated with an overarching pain phenotype. These orphan pain states are often given short shrift because they are not well known. It is often not appreciated that many of these syndromes have surprisingly well characterized animal models. Thus, the human conditions such as erythromyalgia are widely appreciated, but in neuromuscular biology, there are connective tissue disorders (Ehlers-Danlos, Marfan Syndrome, or Bethlem myopathy), in nerve injury there are neuropathies induced by heterologous immune mechanisms secondary to infectious processes (Leprosy, Borreliosis/HIV) (23, 24). Paraneoplastic conditions leading to sensorimotor, autonomic, vasculitic and or motor neuropathies (25) occur in 2–6% of patients with a variety of tumors (lymphoma, non-small cell lung cancer, carcinomas of various organs) (26). These conditions are often associated with strong and unremitting pain states that are not effectively managed with available therapeutics. Understanding these pathologies thus represents a clinical imperative. While many of these have an incidence of 200,000 or less in the US population, study of these syndromes has significant merit. Aside from the evident importance of treating the pain in the suffering patient, investigating these pain phenotypes provides the opportunity for developing novel insights into the processing of pain signaling. The importance of NaV 1.7 arises from the detailed study of the human conditions associated with gain and loss of function in small populations (27). In the “paraneoplastic” syndrome, this diagnosis which may be associated with cancers of virtually every peripheral organ (28). The antibodies developed in the various paraneoplastic states likely differ and if so, then so perhaps does the target engaged leading to the pain states. Such differences provide the ability to assess the role played by these different targets and provide insights into mechanisms which may underlie more common states. We must consider the possibility that a common diagnosis (fibromyalgia, musculoskeletal, paraneoplastic syndrome) may actually reflect a composite of a myriad of biologically different syndromes with difference in underlying therapeutic pharmacology.

## Pain Therapeutics in the Neonate

Classically it was believed that neonates were pain insensate due to the neural development. This assumption and the belief that anesthetics were cardiotoxic led to open heart surgeries in newborns which were performed under nitric oxide and muscle relaxant. The evident success of early trials with fentanyl and volatile anesthetics demonstrated quickly the virtues of adequate anesthetic technique in the new borne (29). While it is clear that regulation of pain in the neonate is critical, given it potential role neural organization, the developing status of the newborn poses issues which are unique to the new born (30). They display unique pharmacokinetics and metabolizing enzymes. Current controversies on the potential effects of volatile anesthesia (31) and spinally delivered drugs (32) emphasize this concern. Our challenge here is to extend validation of the effects of drugs on pain behavior in the preclinical model to that required to manage the human neonatal pain phenotype with appropriate concern for the effects of the intervention in the developing nervous system.

## Pain Therapeutics in Male and Female

The historical perspective of using a single sex to undertake efficacy/mechanistic studies in pain has clearly been superseded by the appreciation that, where the model pain state is independent of anatomy, attention to both sexes is mandated by the agencies which represent both sexes, as well as the ethics of why the research is being accomplished. Aside from such considerations, sex differences are observed clinically and in the animal model, sex differences provide a natural experiment. Defining a therapeutic as having a sex dependent efficacy has practical significance in drug development of pain therapeutics provides a clue shedding light on mechanism. Clinical experience says that many pain syndromes are predominant in one or the other sex (33). The challenge here is to understand why that difference exists. Little more needs to be said to except note that studies involving one sex should require explicit justification.

## Pain Therapeutics in the Veterinary Patient

Veterinarians appreciate that pain management is an important aspect of veterinary practice with companion animals showing the same impact upon the quality of life as seen in humans (34). If one undertakes a PubMed search for osteosarcoma or osteoarthritis in dog over the last 5 years, there were over 700 papers. The challenge here is to address the treatment of these chronic pain states in the companion animal model and promote development of novel pain therapeutics. This charge raises the same issues as do those for human, and if anything poses greater problems related to therapeutic ratios, because of the need to err on the side of higher dosing to assure adequate pain relief in the non-verbal organism (35). It should be appreciated that many drug development protocols require safety assessments in two species, one of which is large animals and not infrequently dogs. Accordingly, implementation of drug development strategies for veterinary pain therapeutics though the FDA Center for Veterinary Medicine, has important benefits for timely advancement of candidates though the proof of concept of target engagement in large preclinical models of pain states which are naturally expressed (e.g., osteoarthritis). Our emphasis here is on the development of improved analgesic regimes and delivery platforms for the veterinary patient.

## Routes of Therapeutic Delivery

Today, we are faced with an exciting variety of routes and platforms to deliver therapeutics to control pain may usefully consider a variety of interventional routes which serve to optimize therapeutic efficacy and utility. We remember that the utility of morphine as an analgesic and cocaine as an anesthetic in fact reflects development of the hypodermic needle and syringe in the 1850's. In the 1970's, the simple expedient formulating extended-release oral morphine formulations allowed BID dosing and arguably represents one of the great advances in the management of chronic cancer pain (36, 37). Today, we can expect a significant contribution to efficacy though implementation of a multiplicity of novel routes and delivery systems. Aside from oral, and IV routes, increasingly sophisticated delivery systems have become relevant with the various formulation/delivery platforms including strategies to enhance transdermal transport (38) and the subcutaneous delivery for biologics (39). Attention to delivery of therapeutics in to the neuraxis has involved the traditional intrathecal and epidural routes with significant attention being paid to delivery strategies (40) and now intraganglionic approaches (41), nasal therapeutic delivery resulting in absorption into the CSF, in part though the cribiform plexus (42) have evolved. The development of formulations that can produce graded release as with nanoparticle, or be manipulated as with magnetic targeting (43) or activation by ultrasound (44) are but several examples where therapeutics can be given properties to alter their distribution and PK. The challenge is to develop strategies to more efficiently deliver the therapeutic to the target (within and outside the neuraxis), have salutary effects upon the PK profile and reduce adverse effects associated with off target interactions. The development of these routes go hand in hand with development of new therapeutic platforms.

## Therapeutic Platforms

A major challenge in developing pain therapeutics is the tailoring of the activity with the variable requirement of the pain to be managed. The history of pharmacotherapy has with few exceptions engaged the interaction of small molecules having agonists, antagonist, or inhibitory effects upon cellular targets. Arguably, nowhere has this been more apparent than in the area of pain therapeutics. Broadly, we might perceive the need to manage pain might be considered to fall into the acute (<24–72 h), transitional (days to weeks) and Persistent (weeks to month or longer). Many of the therapeutic approaches with small molecules involve interventions that are best associated with relatively short-term usage. Of great import has been the development of agents which serve to produce changes which endure beyond their PK by altering cellular function. Targeted toxins such as those focusing on the TRPV1 receptors associated with nociceptive afferents (45) or toxins targeted at the demise of the cell, such as sP-saporin (46) or the releasing function of the nociceptive afferent (47) though targeted uptake though channels and receptors (e.g., Neurokinin 1 receptor) associated with nociceptive afferents or the 2nd order neuron are gaining credibility. These therapeutics offer the possibility of producing long lasting to irreversible changes in pain processing. Such approaches offer the possibility of tailoring the time course of the pain control to the temporal course of the patient's pain. Approaches leading to long lasting but reversible changes serve the need for those intermediate pain states, while permanent cell loss, would likely not be employed other than in the terminal patient. Thus, toxin platforms resulting in persistent but reversible effects such as the botulinum toxins might represent a therapeutic approach for persistent pain states in a non-terminal patient. The use of neuraxial antisense (48) or viral transfection (49) targeting changes in genome to down regulate the expression of a critical element in the pain pathway or increase the expression of an endogenous modulator has great promise to manage persistent pain states Intraganglionic injections have also been suggested as an efficient tool to delivery of therapeutics to alter cell function and genomics of the sensory afferent (41). These approaches have taken surprising turns with the development of the DREAD reagents that allow the selective stimulation of inhibition of cells transfected to express transducers that recognize only a synthetic drug. Here the delivery of the drug leads to regulatory effects only in the transfected cells (50). The ability to produce long-term, regulated changes in processing offer a potential to modify the pathological expression of pain by modifying system function. Here, two important issues relevant to the study of these novel delivery motifs and platforms are demonstrating that the effects are not limited to changes in threshold, but also result in changes in the processing of information relevant to the aversive nature of the pain state. This is particularly true for spinal neuraxial interventions. Strategies such as performing conditioned place preference to show that animals in a chronic pain condition exposed to the therapy may no longer display the place preference for a drug otherwise effective in that pain state. The second issue is therapeutic safety.

## Stimulation

Activation of neural circuits by stimulation applied to the nerve, the dorsal root ganglion, the spinal cord and structures within the brain by deep electrodes have been variously reported to alter pain reports (51–54). Activation of these neural systems has been achieved though programmable systems. Noninvasive modalities including transcranial magnetic stimulation; cranial electrotherapy stimulation, transcranial and direct current stimulation, have been reported and represent intriguing approaches interesting, but thus far of limited utility (55). Experimentally, the development of optogenetic modalities may be considered for achieving selectivity and initiating excitatory or inhibitory effects (56). Systematic studies are required to serve in the development of the therapeutic modalities to demonstrate efficacy and continued utility.

## Mindfulness and Higher Order Integration

Pain is ultimately a subjective experience that reflects on the integration of sensory, cognitive and affective factors in the context of past experiences that represent learned associations and memory. It has become increasingly evident that cognitive interventions such as mindfulness, meditation, expectations, and beliefs defined in well controlled study conditions can impact the pain state reported by the human observer (patient) evoked in experimental or clinical models (57). Considerable attention is being paid to the rigor with which such studies are performed (58). Of note the developing insights provided by the use of electrophysiological and imaging technologies have provided important insights into the covariates that may occur with these cognitive interventions allowing them to be rigorously studied (59). Even preclinical models have suggested that changes in brain activity can be associated with changes in sensory processing (60). This line of research promises to provide exciting insights into underlying mechanism. Do these cognitive interventions display functional specificity (e.g., anxiety produced by a painful stimulus vs. an environmental stressor, e.g., the thump-thump of an approaching helicopter). Is there a pharmacology to mindfulness or placebo that can block or enhance the intervention? Are the changes in higher order responses reflective of changes in ascending afferent traffic? It is readily appreciated that the classical conditioning of a red light after pairing with shock leads to a blood pressure rises that reflect the descending linkage to preganglionic sympathetic and stress can lead to changes in heart rate variability (61). Such work promises to shed light on mechanisms underlying the affective response to the pain state as well point to ways of facilitating the effects of the intervention.

## “Complementary” and “Alternative” Medicine

In the course of considering development of therapeutics, approaches exist which are not considered to be part of the mainstream of a country's own tradition nor conventional medicine and are not integrated into the dominant health-care system, e.g., are referred to as complementary medicine (62).

This field includes the more mainstream and accepted forms of therapy, such as acupuncture, homeopathy, and Oriental practices. These therapies have been practiced for centuries worldwide. Traditional alternative medicine may include various products arising from natural products. Such approaches may arise from rationales that do not match those which arise from conventional wisdom. The NIH created a center for such therapeutic approaches (National Center for Complementary and Integrative health). The use of such therapeutic regimens is part of the published literature and to a certain extent, medical practice (63, 64). The potential value to come from the unexpected cannot be discounted and should be considered to be appropriate subject matter for investigation. Such work is amenable to research as the scientific method is agnostic. Providing one can establish an appropriately defined dependent and independent variables and perform rigorously controlled experiments, as we demand of any preclinical or preclinical trial such research would be welcomed.

## Therapeutic Tolerability/Safety

As noted above, failure of a pain therapeutic, often hinges on limited efficacy and adverse event profile. Preclinical development of analgesic therapeutics with novel targets must bring forth the data that substantiates a significant therapeutic ratio. The challenges here are to determine the relative dosing margin that separates the readout that define changes in specifically in pain behavior and the adverse or countering behaviors (65). It often seems that while the assessment of pain behavior in published work may be reasonably sophisticated, it often appears to be the case that the assessment of the side effect profile is relatively incomplete. At the early phases of therapeutic development, side effects maybe classified in terms of changes in motor function or activity which provide a demonstration that changes in pain behavior are selective for pain vs. a general behavioral suppression or the generation of competing behaviors. It is an oft told story that prefrontal lobotomies prevented the affective component of the pain state (It hurts, but it does not bother me?). Yet, the loss of the affective dimension was said often to come hand in hand with some changes in affect with motivation (66). This is not at present an issue with conventional drug targets or after spinal delivery. The challenge must be to develop sufficient information to be able to specify the minimum dose necessary to produce significant analgesia vs. the minimum dose required to produce significant adverse signs, e.g., the therapeutic index. A systematic assessment of therapeutic ratios and maximum achievable effects on behavioral endpoints and the relationship of these endpoint to clinical efficacy remains to be systematically accomplished on a broad scale. An additional note must reflect upon issues of safety. Preclinical studies clearly assess the therapeutic ratio for adverse events. The use of neuraxial therapeutics pose an additional burden, where the high concentrations and extended exposure or the evident effect upon cell function (toxins, viral transfection) may incur risks of systems damage. As an example NK1 targeted toxins (sP-Saporin) is noted to kill NK1(+) cells, but the examination of this loss of a small population of dorsal horn neurons was unaccompanied by additional signs of tissue morbidity (67). We must imagine that studies examining such toxins/transfection agents must similarly be prepared to describe the consequence to local cell system, if any (68). It would be difficult to assert that the effects of neuraxial therapeutic that leads to profound neuroinflammation owes its effects uniquely to the effects observed on the targeted cell.

## Therapeutics and Abuse Liability

Aside from limited efficacy, nothing has driven the current emphasis upon developing new therapeutic interventions more than the opiate abuse. As discussed above, this multifaceted phenomenon reflects the biology of reward (positive reinforcement) and dependence/withdrawal (negative re-enforcement). We must recognize that the development of nonopioid analgesics does not do away with the risk of abuse potential by therapeutics. This problem may well surface with the development of drugs which alter the pain response by virtue of an effect upon higher order function. It is a historical observation that amphetamine has pain reliving properties in humans (69) and more recently alkaloids found in the Khat tree leaves (70). The challenge for those agents targeting higher order function is to define drug targets that do not lead to a positive re-enforcement in the absence of a pain state.

## Measured Preclinical Endpoints Defining Analgesic Therapeutic Efficacy

Pain is formally described as “An unpleasant sensory and emotional experience associated with, or resembling that associated with, actual or potential tissue damage,” Analgesia is variously described as an insensibility to pain without loss of consciousness. An important challenge in the development of a pain therapeutic in which we manage not only the nociceptive aspects of the stimulus event leading to physiological changes in function that are reflected in the autonomic and hormonal aspects of the stress response but as well, the reduction in the aversive properties of the stimulus condition (as defined by the behavioral response of the organism). Historically this dichotomy has been reflected in the appreciation of the sensory/discriminative vs. affective motivational aspects of the pain response. The preclinical assessment of efficacy has usefully employed threshold measurements to define target engagement leading to a physiological effect. There is a reasonable assumption of the therapeutic significance of interventions which alter thresholds (with the caveat that the changes are not secondary to an effect upon the ability of the animal to respond or obstruct though confounding behaviors the evoked response). The significance of this observation to the pain construct (e.g., “An unpleasant sensory and emotional experience…..”) is contingent upon the assertion that the change in threshold represents changes in the information content of the message provided to supraspinal integrative centers. Confirmation of this covariance would at some point require demonstration that the intervention changes not only the threshold but alters the aversive quality of the stimulus condition. Of note we do recognize that interventions which alter complex, supraspinally mediated stimulus evoked response patterns (like hind paw thermal stimulation evoking a complex response phenotype-hind paw licking- or weight bearing) clearly provides evidence that the intervention has altered supraspinal trafficking that leads to the supraspinally organized response to that input. In addition, we would consider it advantageous to directly assess if the aversiveness/negatively rewarding state generated by the pain conditions has been removed. Several ways are available. One might assess the pairing of the drug delivery with a novel stimulus environment, as with the conditioned place preference paradigm (71) or some reappearance of some normal behavior otherwise suppressed by the stimulus condition (e.g., spontaneous activity, marble burying, nest building) (72). We consider these endpoints (threshold changes and normalization of stimulus evoked behavioral suppression) to be crucial criteria for interventions defined in preclinical models that are asserted to have “analgesic” properties.

## Data Sets for the Therapeutic Drug Studies

This challenge is only a challenge because it is not so often done and that is for the author to provide information that substantiates a rigorous study and a rigorous analysis of the data set provided by the study to permit others to draw comparisons. Several points can be considered based on the ARRIVE guidelines (73, 74) the study should provide: (i) details of the power analysis undertaken a priori to estimate group size (75); (ii) define statistical analysis and justification for selection of tests (e.g., parametric vs. non parametric assumptions); (iii) indicate measures to reduce bias including random allocation and blinding, and how they were performed; (iv) provide graphics that displays individual data (e.g., scatter plots); (v) define and justify metrics employed (e.g., raw thresholds/latencies; differences from baseline; % of baseline, % Maximum possible effect, etc.); (vi) where possible provide an active control of a common drug (e.g., gabapentin, morphine, ibuprofen) with which the comparisons of novel test article can be compared across laboratories.

## Clinical Translation

Efforts to define efficacy in the human patient is the crucial step in moving a therapeutic intervention, either pharmacologic or non-pharmacologic, into human application. Experimental studies to demonstrate effects of target engagement in the human population involving experimental and clinical pain represents a large field of endeavor providing insights with rigorously controlled assessments (76, 77). The evolution of such assessment to the current emphasis upon randomized, controlled, and blinded studies has provided the basis for maximizing the inferential capabilities of such trials though rigorous efforts to minimize the multiple sources of study bias. The recent series of papers based on ACTTION work (Analgesic, Anesthetic, and Addiction Clinical Trial Translations, Innovations, Opportunities, and Networks) provides an important basis for defining the issues that refine the assessment of analgesic therapeutics in the human condition (78). Of particular importance from the perspective of the present journal is adherence by its accepted manuscripts to the guide lines for what constitutes robust clinical trials laid out in the CONSORT (Consolidated Standards of Reporting Trials) check lists to provide for complete and unbiased reporting of trial methods and results (79). The development of novel therapeutic targets, formulations and delivery platforms and particularly in the case of interventional modalities (stimulation, infusion systems) (80) pose significant challenges where long term changes might be anticipated. Characterization of the contribution of behavioral interventions such as meditation, mindfulness, hypnosis and placebo are clearly issues requiring diligent consideration in their being submitted for systematic clinical trial evaluation (58). Characterizing treatment effects in terms of reduced consumption of ancillary medication, establishment of the appropriate comparator groups and the role of subject stratification given the appreciation of the complexity of even commonly diagnosed pain phenotype all pose significant challenges that the Journal invites for detailed consideration.

## Conclusion

Developing interventions which selectively alters the processing of pain relevant sensory information with no changes in normal sensory-motor function presents a daunting challenge. I would conclude by emphasizing several specific issues

(i) Much work is targeted on the large populations suffering from pain which is persistent. These individuals present problems that must be targeted, as the pain as its impact upon quality of life is chronic. Particular challenges however are those patients which receive transient injuries/interventions and then for reason that are not clear progress to a state of persistence. Preventing that shift must be considered a real challenge.

(ii) The focus on problems faced by a large part of the populations is rational, however we need to draw focus to those human pain conditions which are perhaps rare but deserve our attention. Not incidentally, addressing those pain states provide insights into comparative changes that provide insight for all pain states. It is recognized that the importance of NaV channel subtypes arose from small genetically related families with altered pain phenotypes which led to the recognition of the role of NaV1.7 and to the development of NaV targeted therapeutics.

(iii) Patient sex, and age (e.g., the neonate), are patient characteristics that separate them from the population at large and which until recently was less rigorously studied and yet in whom the need for pain management is critical.

(iv) I strongly emphasize the need for developing veterinary pain therapeutics. It is a natural accompaniment to the use of these animals in developing the human therapeutic and it provides ancillary validation of target engagement and efficacy in natural clinical models.

(v) Great advances have arisen in the development of therapeutic targets and development of novel platforms and delivery routes. These, toxins, AAV's and ASO's offer the ability to specifically target critical links in the neuraxial pathway. Importantly they offer three added advantages. (1) they can move target manipulation rapidly from structure/sequence to structure engagement. (2) The platforms offer the possibility of producing interventions that may be relatively short lasting to irreversible and with changes in between to tailor the intervention to the time frame of the pain state, and (3) it is clear that pain states are complex, At present a cancer patient may routinely be on 3–4 pain meds, not because the pain meds are in effective, but because the pain states have overlapping mechanisms with distinct pharmacologies. Developing small molecules to engage any one target may require years not to mention three or more targets. With ASO/AAVs, multiple targets can be concurrently engaged.

It is clear that the future holds many promises to reach the goal of pain management. The tools are at hand to be grasped.

## Author Contributions

The author confirms being the sole contributor of this work and has approved it for publication.

## Conflict of Interest

The author declares that he sits on several advisory boards for pain therapeutics. This review and commentary was conducted with a strong effort to avoid commentary that could be construed as reflecting a specific bias representing conflict of interest. His potential conflicts are on file with the journal office.
